# Robotically assisted internal mammary artery harvesting—will single-port systems be useful?

**DOI:** 10.1093/icvts/ivae107

**Published:** 2024-06-06

**Authors:** Johannes Bonatti

**Affiliations:** Department of Cardiothoracic Surgery, University of Pittsburgh School of Medicine and UPMC Heart and Vascular Institute, Pittsburgh, PA, USA

**Keywords:** Robotic surgery, Internal mammary artery harvesting, Multiport, Single port, Minimally invasive coronary artery bypass grafting

I read with great interest the experimental work by Stein *et al.* which looked at bilateral internal mammary artery (IMA) harvesting using the da Vinci SP single-port surgical robot [1]. The authors conducted this study in 2 human cadavers and in 2 live pigs. They used a left-sided subcostal incision for robot docking and managed to harvest the IMA bilaterally in all experiments. The docking process took between 14 and 21 min and bilateral IMA harvesting could be accomplished in a time frame between 65 and 125 min. The authors conclude that they demonstrated feasibility of the robotic single-port approach and state that harvesting was technically less demanding than through a mini-thoracotomy.

The da Vinci SP system is one of the latest iterations of the first robot ever used for coronary bypass surgery. Whereas in the classic robotic IMA harvesting procedure [2] an 8- or 12-mm camera port and two 8-mm instrument ports were inserted into the chest the single-port version uses a 25-mm tube with 4 channels that accommodate an articulating camera and up to 3 channels for robotic instruments which are also flexible. This flexibility probably supported the successful conduct of the current IMA harvesting experiments. As expected the harvesting process was more difficult at the caudal end of the left-sided IMA as the single port was located parallel to the vessel course. Another challenge was adjustments of the SP system remote centre during the procedure and a software solution for this problem in the future is projected. The harvesting times achieved are encouraging and I am sure appropriate times can be achieved in the clinical setting.

Exploring the utility of the da Vinci SP system for internal mammary artery harvesting was certainly a necessary step and it was good to see that a European group of researchers after many years of slow development in robotic cardiac surgery and recent renewed interest [3] took on this project. Concerning the conclusion that the robotic SP approach was easier than bilateral IMA harvesting through a mini-thoracotomy I would like to state that the latter is probably a technically too demanding endeavour anyway, especially in obese patients. I would also like to ask the question why given the fact that robotic technology has existed for >25 years surgeons are so reluctant to even pursue robotic graft take down. The main advantage of robotic IMA harvesting is that the surgeon is fully immersed into the chest cavity and can harvest the internal mammary arteries in their full length. In the classic minimally invasive direct coronary artery bypass grafting (MIDCAB) [4] procedure or the corresponding minimally invasive cardiac surgery—coronary artery bypass grafting operation (MICS-CABG) [5] for multivessel disease the internal mammary arteries are harvested through the minithoracotomy using special spreaders or lifting devices which are relatively traumatic and the view on the graft, specifically the left IMA, is tangential. For both the left and right internal mammary reaching the distal end can be an issue and too short a graft length is a potential complication.

Concerning the single-port principle in general it needs to be discussed whether a 25-mm port incision is better than three 8-mm port holes in classic robotic IMA harvesting. With three small incisions the trauma is dispersed, pain may be distributed across the chest, and the question remains whether herniation of underlying content is possible through the single-port incision. At least proper port hole closure needs to be guaranteed.

As can be read from the paper the next step is probably clinical application of the SP system for IMA harvesting in a MIDCAB operation. The authors mention that introduction of a coronary artery stabilizing device through the subcostal incision should be possible. I agree with this statement but would like to encourage the group to look beyond the MIDCAB procedure and to develop a totally endoscopic coronary artery bypass grafting operation [6] in the models described. Totally endoscopic coronary artery bypass grafting is one of the few minimally invasive procedures in heart surgery that only work using robotic technology. Attempts to perform these operations with standard thoracoscopic instrumentation have essentially failed [7]. The surgical robot, however, is a beautiful, sophisticated tool that allows for endoscopic suturing of vascular and micro-vascular anastomoses. The community of minimally invasive heart surgeons should definitely set the goal at developing totally endoscopic coronary artery bypass grafting and take advantage of current new developments in robotic technology to achieve this goal.
